# Mechanisms Linking Obesity with Non-Alcoholic Fatty Liver Disease (NAFLD) and Cardiovascular Diseases (CVDs)—The Role of Oxidative Stress

**DOI:** 10.3390/cimb47090766

**Published:** 2025-09-16

**Authors:** Fani-Niki Varra, Michail Varras, Viktoria-Konstantina Varra, Panagiotis Theodosis-Nobelos

**Affiliations:** 1 Medical School, Democritus University of Thrace, 68100 Alexandroupolis, Greece; 2 Department of Pharmacy, School of Health Sciences, Frederick University, Nicosia 1036, Cyprus; 3 Fourth Department of Obstetrics and Gynecology, ‘Elena Venizelou’ General and Maternity Hospital, 11521 Athens, Greece; 4 Department of Pharmacy, School of Health Sciences, University of Patras, 26504 Patra, Greece

**Keywords:** obesity, hyperlipidemia, NAFLD, oxidative stress, endothelial cell function

## Abstract

Obesity concerns a wide range of the population, tending to become a major factor for diseases’ progression and fatality rate increases, with implications concerning the cardiovascular system’s deterioration. Obesity is closely linked with metabolic derangements concerning lipid storage and circulation, and the cellular metabolism affecting most of the internal organs, especially liver and cellular function. In this current study, an analysis of the linking mechanisms between obesity, lipid deterioration, liver, and lipid tissue homeostasis will be performed, with special attention to the pathophysiological characteristics of these detrimental effects on the NAFLD (non-alcoholic fatty liver disease) and the cellular function of the endothelial blood cells, with special reference to the additional burdening of obesity on the autonomous nervous system signaling, and the resulting hypertension. Despite the very complex and pluripotent pathogenic mechanisms with which obesity is intervening in these processes, it could be safely deduced that metabolic and lipid transport manipulation could serve as a crucial factor towards the cellular and tissue function improvement, as the interlinkages in the mechanisms, although highly analyzed, have not been completely deciphered until now.

## 1. Introduction

Obesity is a significant global public health crisis in the 21st century, with its prevalence rising steadily across both developed and developing nations. As a multifactorial condition characterized by excessive adiposity, obesity is strongly linked with a number of various metabolic, hepatic, and cardiovascular disorders. Among these, non-alcoholic fatty liver disease (NAFLD) and cardiovascular diseases (CVDs) stand out as major causes of chronic illness and premature death [1,2]. NAFLD is considered to be the hepatic component of the metabolic syndrome and includes a continuum from benign hepatic steatosis (fat accumulation in the liver) to non-alcoholic steatohepatitis (NASH), a condition with inflammation and liver cell damage. This can lead to hepatic fibrosis (hepatic scarring), cirrhosis (severe hepatic scarring), and then hepatocellular carcinoma, a type of liver cancer [3]. In parallel, obesity markedly increases the risk for developing CVDs, such as atherosclerosis, myocardial infarction, and heart failure, primarily through its adverse effects on systemic inflammation, impaired insulin sensitivity (insulin resistance, IR), and abnormal lipid metabolism [4].

At the molecular level, obesity triggers a series of metabolic and immunological disturbances that underpin both NAFLD and CVDs. Adipose tissue, particularly visceral fat, becomes dysfunctional and secretes pro-inflammatory cytokines, including tumor necrosis factor-alpha (TNF-α), interleukin-6 (IL-6), and resistin, which promote hepatic fat accumulation, endothelial dysfunction, and vascular inflammation [5]. The insulin paradox in the pathogenesis of NAFLD refers to a tissue-specific paradox, where insulin in adipose tissue plays a central role by exacerbating peripheral lipolysis and free fatty acids’ (FFAs) transfer to the liver, while it fails to suppress de novo lipogenesis in the liver, thus fostering hepatic steatosis and promoting atherogenic dyslipidemia [6]. Additionally, oxidative stress and impaired mitochondrial function further contribute to hepatocellular damage and cardiovascular remodeling [7].

Given the overlapping pathophysiological pathways, understanding the molecular mechanisms, especially those implicating lipids’ deterioration, linking obesity, NAFLD, and CVDs, is crucial for the development of integrated therapeutic and preventive approaches. This literature review aims to clarify the key molecular and pathological interconnections between these conditions, highlighting their clinical implications and future research directions.

## 2. Materials and Methods

An electronic literature search was performed on the PubMed database and the Google Scholar database to identify relevant peer-reviewed articles published up to 31 August 2025, without time limit. The search has been restricted to the availability of the full article version, randomized-controlled trials (RCTs), systemic reviews and meta-analyses (SRMAs), cohort studies, and narrative reviews. Excluded studies were case reports, editorials, and animal experiments. A search was performed on PubMed and Google Scholar with the specific keywords: “obesity”; “non-alcoholic fatty liver disease”, or “NAFLD”, or “metabolic-associated steatohepatitis”, or “non-alcoholic steatohepatitis”, or “NASH”, or “metabolic-associated fatty liver disease”, or “MAFLD”, or “metabolic-associated steatohepatitis, or MASH”, or “liver steatosis”, or “liver fibrosis”, or “liver cirrhosis”; “cardiovascular diseases” or “CVDs”; hypertension or “blood pressure”; pathophysiology” or “pathogenesis”; “oxidative stress”; “lipotoxicity”; and “endoplasmic reticulum stress”. The selection process involved assessing titles, abstracts and full texts to make sure they fulfilled the predefined inclusion and exclusion criteria and for the quality check. Also, the reference list of previously identified relevant studies was thereafter manually examined to find additional related publications.

## 3. Lipid Derangement and NAFLD

NAFLD is a heterogeneous liver disease, which ranges from liver accumulation of lipids (steatosis) to non-alcoholic steatohepatitis (NASH) [8]. The American Association for the Study of Liver Diseases (AASLD) defines NAFLD as (i) primary hepatic steatosis confirmed by imaging (such as ultrasound, MRI, or CT scan) or liver histology (biopsy), and (ii) exclusion of secondary hepatic fat accumulation, such as significant alcohol consumption, prolonged use of steatogenic medications or specific hereditary medical disorders that also lead to hepatic fat accumulation [9]. The diagnosis of hepatocellular steatosis requires the ectopic lipid deposition into hepatocytes to exceed 5% of the total liver weight. Hepatocellular steatosis may progress to the more severe form named NASH [10]. NASH is defined as the presence of at least 5% primary hepatic steatosis along with various degrees of liver cell inflammation and damage, which can be confirmed with a liver biopsy. NASH may progress through the stages of liver disease, leading to advanced fibrosis, cirrhosis, and eventually hepatocellular carcinoma (HCC) [10]. The high frequency of concurrent primary hepatic steatosis in NAFLD alongside obesity, hyperlipidemia and type 2 diabetes mellitus (T2DM) strongly supports the premise that NAFLD is the hepatic representation of the metabolic syndrome [9,11].

**Figure 1:**
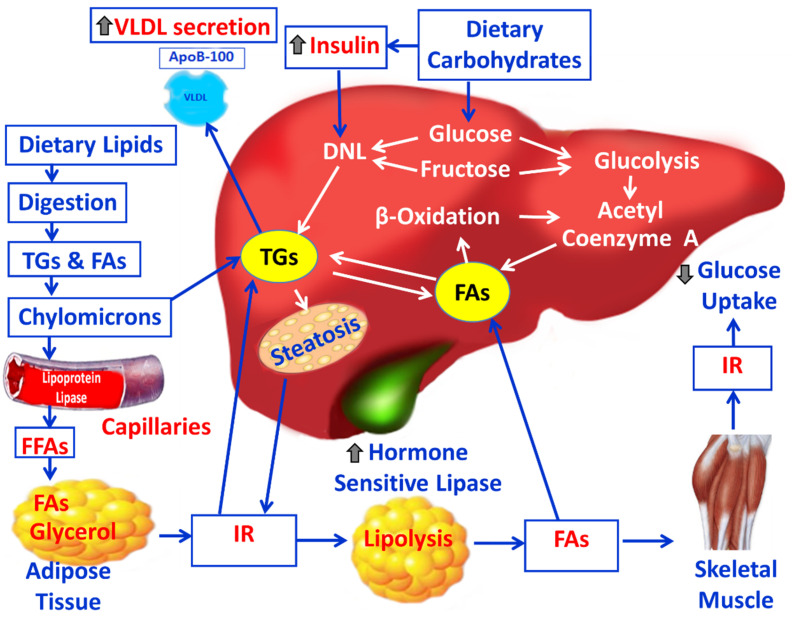
The metabolism of TGs and carbohydrates in the liver and their implication in metabolic abnormalities are illustrated.

Triglycerides (TGs), also called neutral fats, triacylglycerols, or triacylglycerides, are the major vegetable and animal fats from diet (exogenous TGs) and are the main form of fat stored in adipocytes of the human body. The plasma total TG concentration is determined to identify metabolic disorders like metabolic syndrome, which increases the risk of CVDs and insulin resistance (IR). TGs are hydrolyzed in the gut by lipases releasing fatty acids (FAs) and monoglycerides through lipolysis. Only short- and medium-chain FAs (≤12 carbons) are absorbed directly by enterocytes and then transferred into the bloodstream by serum albumin. In contrast, long-chain FAs (>12 carbons) are re-esterified into TGs within the enterocytes and are transported by lipoproteins through the body [12]. Lipoproteins are classified by density into different categories including ultra low-density lipoprotein (ULDL), very low-density lipoprotein (VLDL), low density lipoprotein (LDL) and high density lipoprotein (HDL) [12]. Lipoprotein lipase (LPL) is an extracellular enzyme, anchored to the surface of vascular endothelial cells, where it cleaves TGs into free fatty acids (FFAs) that are readily absorbed and utilized by surrounding cells, such as muscle and adipose tissue, for energy or storage [12]. The monoglycerides in the smooth endoplasmic reticulum (ER) of the enterocytes are re-esterified to triglycerides, which are packaged into chylomicrons for transport in the blood. Chylomicron remnants are cholesterol-enriched lipoprotein particles formed when large, TG-rich chylomicrons deliver their FAs to the peripheral tissues [13]. They are then taken by the liver via receptor-mediated endocytosis. Within hepatocytes, these remnants undergo intracellular processing, which includes the liberation of FAs [13]. The liver and adipose tissue are the main sites of endogenous synthesis of TGs. Also, carbohydrates from the diet (e.g., glucose and fructose), are converted into FAs and triacylglycerols (TAGs) in the liver through a process called de novo lipogenesis (DNL) [13]. Hepatic de novo synthesized TGs are either stored in intracellular lipid droplets (LDs) for later use or packaged into VLDL particles and secreted into the plasma [13,14] (Figure 1). Apolipoprotein B100 (ApoB100) is the critical structural protein that is essential for the assembly and secretion of VLDL-TG particles from the liver [14]. Once FFAs are taken up by cells or synthesized by de novo lipogenesis, they are transported by intracellular fatty-acid binding proteins (FABPs) to the hepatic mitochondria. Inside the mitochondria, FFAs undergo beta-oxidation to produce ATP, which supports cellular and tissue homeostasis [12,15]. The acetyl-CoA produced from the β-oxidation of FAs in the liver mitochondria is converted into ketone bodies, i.e., acetoacetate, beta-hydroxybutyrate (BOH), and acetone during the aerobic respiration. This process, known as ketogenesis, provides an alternative energy source for tissues, such as the brain and the heart [15]. Primary sources of FAs are (i) dietary lipids, (ii) endogenous hepatic de novo synthesis from non-lipid precursors and (iii) adipose tissue-derived FAs from circulating non-esterified fatty acids (NEFAs) [13]. Also, FAs are important components of all cellular membranes, forming the hydrophobic “tails” of phospholipids, which are the primary building blocks of cell membranes. In adipose tissue, FAs are re-esterified into TGs in lipid droplets for energy storage [12,16]. During fasting or prolonged exercise, the hormone-sensitive lipase (HSL) is activated by hormones like glucagon and adrenaline, breaking down TGs in adipose tissue to release FFAs for energy. Conversely, insulin inhibits HSL, suppressing lipolysis and promoting fat storage when energy is abundant. This ensures a steady supply of fuel to skeletal muscles and the heart muscle, independent of consuming food [17,18]. FFAs are non-esterified fatty acids that circulate in the plasma. FFAs are primarily bound to serum transport proteins, like albumin in the bloodstream. Increased dietary intake of FAs, combined with excess adipose tissue and IR, promote adipocyte lipolysis, releasing more FAs into the blood. These circulating FAs then travel to the liver, increasing hepatic lipid levels. This leads to an accumulation of TGs in hepatocytes and prompts the liver to package these triglycerides into VLDL particles for export, resulting in increased VLDL overproduction and associated dyslipidemia [13]. 

### 3.1. Pathophysiology and Mechanisms of NAFLD and the Impact of OS

**Figure 2:**
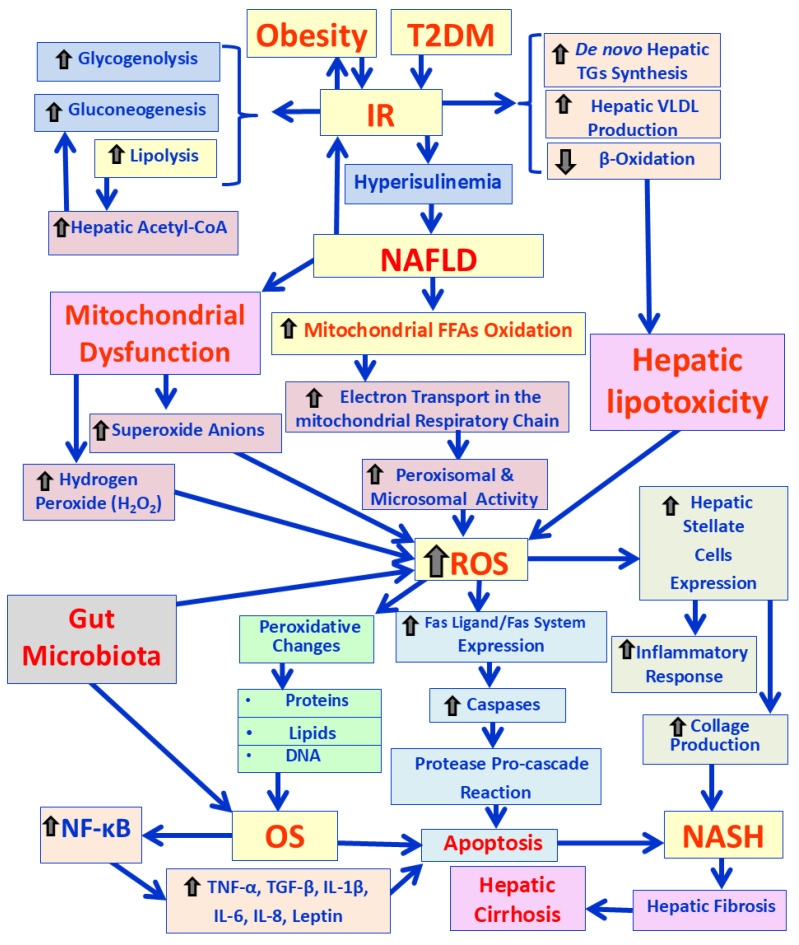
Schematic presentation of the pathogenetic mechanisms involved in the development of NALFD and NASH.

The pathogenesis of NAFLD is a complex multifactorial disease. NAFLD pathogenesis is considered a complex disease that is caused by many contributing factors. The traditional “two-hit model” is now considered outdated and insufficient. The “first hit”, as the initial stage, according to the “two-hit model” of NAFLD pathogenesis, is characterized by concomitant (i) increased hepatic steatosis, and (ii) IR [19]. OS is the initiator of the “second hit” [20]. A large number of adipokines, such as leptin, adiponectin and resistin, regulate FFAs to induce reactive oxygen species (ROS)-mediated injury to the liver [21,22]. However, the traditional “two-hit model” is now replaced by the “multiple-hit model”, implicated in the pathophysiology of NAFLD. This “multiple-hit model” suggests a complex interplay between several interaction mechanisms, which occur in parallel. These mechanisms include: (a) genetic predisposition, (b) various lifestyle risk factors (e.g., poor diet, rich in fructose, saturated fats and calories, smoking, and sedentary behavior), (c) metabolic deregulation (e.g., hyperglycemia, IR, dyslipidemia and lipotoxicity) and (d) alterations in gut microbiota or dysbiosis [23,24,25]. Lipid accumulation in the hepatocytes increases mitochondrial fatty-acid oxidation capacity and increases electron transport in the mitochondrial respiratory chain, stimulating peroxisomal and microsomal activity, which is associated with increased ROS formation. This excess in ROS, in turn, drives OS, damaging cellular components and triggering inflammatory responses, ultimately contributing to the progression of NASH to liver fibrosis, cirrhosis and cancer (HCC) [26]. Additionally, there is a strong connection between hepatic lipid storage and impaired hepatic mitochondrial function [27]. Altered mitochondrial function directly leads to enhanced ROS formation by the organelle itself, and particularly from its ER surface [28]. If electron flow is interrupted at any point in the respiratory electron transport chain, the previous respiratory intermediates can transfer electrons to free oxygen molecules to produce superoxide anion radicals and hydrogen peroxide (H_2_O_2_) [29]. Then, superoxide anion radicals and hydrogen peroxide (H_2_O_2_) induce ROS production. It has been found that ROS generation is associated with APO-1 activation [30] initiating a caspase cascade, which leads to aggregation of inactive pro-caspases. This aggregation causes pro-caspases to cleave and activate each other, creating a “pro-cascade reaction”. Then, these activated initiator caspases activate downstream executioner caspases, resulting in cellular disorganization and apoptosis [22]. Additionally, high levels of ROS can cause oxidative modifications to cellular components, including proteins, lipids and DNA, which further promote OS and cell death [31]. Therefore, mitochondrial dysfunction significantly contributes to the pathogenesis of NAFLD and NASH [32]. Moreover, ROS and lipid peroxidation products activate hepatic stellate cells (HSCs), causing them to secrete excess collagen, a key process in hepatic fibrosis [33]. The hepatic fibrosis worsens when obesity is associated with increased secretion of several inflammatory markers. Studies demonstrate that hepatic steatosis leads to activation of the NF-κB signaling pathway. The activation of NF-κB induces the release of inflammatory mediators such as TGF-β, Fas ligand, TNF-α, leptin, IL-1β, IL-6 and ΙL-8, which contribute to the development of ROS-mediated hepatocellular injury [34,35]. In addition, a liver with steatosis is more vulnerable to inflammation because excessive fat triggers OS by increasing ROS production and overwhelming the body’s antioxidant defenses [36]. Lower adiponectin levels in patients with NAFLD are consistently associated with more severe hepatic fibrosis, including the progression to NASH and eventually cirrhosis [37]. Finally, alterations in gut microbiota or dysbiosis play a large role in the pathogenesis of NAFLD and NASH in obese patients through different pathways, including their influence on energy storage, lipid metabolism, ethanol production, immune balance and inflammation [35]. It has been found that changes in gut microbiota homeostasis induce intestinal inflammation, which can increase intestinal permeability, thus promoting the translocation of bacterial endotoxins into systemic circulation and facilitating the hepatic endotoxemia and low-grade intestinal inflammation [38]. Therefore, gut microbiota alterations are strongly linked to overproduction of ROS and consequent OS, which are responsible for the development of NAFLD and NASH [39] (Figure 2).

### 3.2. Obesity, Lipid Derangement, OS and CVDs

Dyslipidemia, or hyperlipidemia, describes an abnormal amount of serum lipids that increase the risk of CVDs and heart stroke [40]. The atherogenic dyslipidemia consists of increased circulating TGs and FFAs, decreased HDL and normal or slightly increased LDL with increased sdLDL. Elevated levels of plasma apolipoprotein (apo) B are usually the result of hepatic overproduction of apo B containing lipoproteins [41]. Diagnostic criteria for obesity-related dyslipidemia are defined as HDL < 1.0 mmol/L (40 mg/dL) in men and <1.3 mmol/L (50 mg/dL) in women, or blood TGs > 1.7 mmol/L (150 mg/dL) [41,42]. High levels of TGs and low levels of HDL are major risk factors of atherosclerosis by a buildup of plaques inside the large and medium-sized arteries, which are deposits of fatty materials.

In obesity, hypertrophied adipocytes lead to a chronic inflammatory state in adipose tissue, characterized by the infiltration of immune cells and the release of pro-inflammatory mediators, such as IL-6 and TNF-α, contributing to systemic inflammation. This occurs because larger adipocytes experience hypoxia or reduced oxygen, which triggers inflammation [43]. It has been found that FAs in hypertrophic adipocytes trigger specific serine-kinases that attenuate insulin receptor function. Fatty tissue IR consequently causes fatty tissue to release excessive FFAs into the blood circulation, causing accumulation of TGs in tissues like the liver and skeletal muscles, where they normally contain small amounts of fat [44]. Also, obesity-induced hepatic IR is characterized by an inability for insulin to inhibit glucose output [45]. Hepatic IR leads to hepatic endoplasmic reticulum (ER) stress, ROS generation, and inflammation [45]. These processes stimulate gluconeogenesis and lead to hyperglycemia and consequent hyperinsulinemia [46]. Mechanisms that also connect obesity with IR, dyslipidemia and CVDs and stimulate ROS production in the vascular endothelial cells include: (i) reduced levels of HDL; (ii) enhanced clearance of HDL; (iii) high post-prandial TG values; (iv) elevated plasma levels of VLDL; (v) elevated plasma levels of TGs; and (vi) elevated Ox-LDL levels [47].

Elevated levels of plasma TRLs (triglyceride-rich lipoproteins) are due to their delayed hepatic clearance as a consequence of IR leading to TGs accumulation in arteries, promoting macrophage foam cell formation and inflammation [48]. Under these circumstances, CETP (cholesteryl ester transfer protein) activity facilitates the transport of CEs and TGs between HDLs, TRLs and B-lps (apoB-containing lipoproteins) [49] leading to low levels of HDLs and the generation of sdLDL, which are the key components of MetS [50]. Low levels of HDL are associated with reduced FC (free cholesterol or unesterified cholesterol) efflux from macrophage-derived foam cells in the arteries, aggravating atherosclerosis, and are therefore a health hazard for CVDs [48]. Also, the high levels of LDLs are involved in the pathogenesis of atherosclerosis and LDL remains the primary therapeutic target for preventing CHD [51]. sdLDs are more pivotal for atheromatic plaque formation and progression than the larger, lighter LDL particles, because circulating sdLDLs undergo multiple atherogenic modifications in blood plasma, making them strong inducers of inflammation, resulting, therefore, in atherosclerotic plaques formation. These modifications include desialylation, glycation and oxidation [52,53]. Also, sdLDL particles have a reduced affinity for the LDL receptors and a lower catabolic rate. Moreover, sdLDL have a greater susceptibility to oxidation and a higher concentration of PUFAs (rendering their oxidized forms to have greater affinity for the scavenger receptors of macrophages, giving further rise to the inflammatory atherosclerotic lesions). In addition, desialylation of sdLDL particles increases their affinity to proteoglycans in the arterial wall and therefore exhibits greater permeability in the endothelium of the arterial walls [54,55]. Apart from all the above mechanisms, sdLDL cholesterol is able to increase the atherogenic effect by regulating the activity of gene networks, monocytes and enzymes [55].

**Figure 3:**
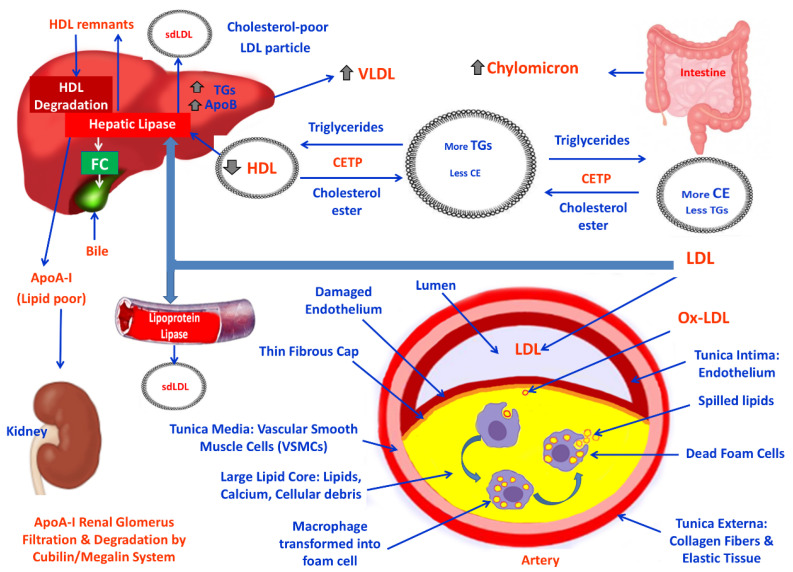
Schematic presentation of the pathophysiological mechanisms involved in atherogenic dyslipidemia in obesity.

Adipo-IR causes increased levels of FFAs. Elevated FAs flux to the liver induces the assembly and secretion of VLDL resulting in HTG (hypertriglyceridemia) [56]. In blood circulation, CETP catalyzes the exchange of TGs from TRLs, such as VLDL, to HDLs and simultaneously transfers CEs from HDL to VLDL. This exchange results in CE depletion and TG enrichment of HDL. HDL containing accumulated TGs serves as a substrate for both LPL and HL. HL modifies TG-rich HDL particles, leading to the release of lipid-poor apoA-I and the formation of smaller, dense HDL remnant particles. Apo-I is freely filtered by the renal glomerulus, then rapidly reabsorbed and degraded by the cubilin/megalin receptor system in the proximal tubule (Figure 3). HDL remnants can bind to specific hepatic receptors. This binding to certain receptors mediates the internalization and catabolism of these remnant HDL particles, decreasing the availability of HDL for reverse cholesterol transport. Moreover, the interchange between TRLs and HDL is mediated by PLTP (phospholipid transfer protein), which transfers surface PLs from TRL to HDL during lipolysis. IR is associated with TG enrichment of HDL particles, elevated HL activity, and the subsequent decrease in HDL levels. Then, LDLs, with the action of HL, generate the sdLDL [48,54,57]. There is an inverse relationship between plasma triglycerides secreted either from the liver (VLDL) or intestine (chylomicrons), and the levels of HDL. Higher levels of TG-rich VLDLs and chylomicrons are correlated with lower levels of HDL. Low levels of HDL are associated with a reduced ability to remove cholesterol from macrophage foam cells, resulting in cholesterol accumulation in the arterial walls, worsening atherosclerotic plaque buildup and increasing the risk of CVDs. Also, high levels of LDLs are responsible for the pathogenesis of CVDs [56]. Moreover, sdLDLs are atherogenic and increase CVD risk [56]. Endothelial dysfunction allows LDL particles, which are rich in cholesteryl esters (LDL-c), to enter the tunica intima of the arteries. Blood monocytes enter the arterial intima, where they are exposed to free radicals that oxidize LDLs, which they then engulf to become lipid-laden macrophages (foam cells) that drive the inflammatory process. Foam cell necrosis in atherosclerotic plaques leads to the accumulation of dead cells and lipids that form a central necrotic core. Over time, this necrotic core grows, and the plaque accumulates calcium, SMCs, collagen, and foam cells, creating a complex lesion that can lead to plaque rupture and cardiovascular events [58]. A typical adverse plaque also known as thin-cap fibroatheroma (TCFA) is characterized by macrophage infiltration, a large lipid-rich core, and a thin fibrous cap. The microcalcifications further destabilize the plaque. The intraplaque angiogenesis within the necrotic core of an atherosclerotic plaque is stimulated by hypoxia and inflammation. This intraplaque angiogenesis is thought to be immature and contribute to plaque instability, inflammation, intraplaque hemorrhage and thrombus formation on the vascular wall. This thrombus can grow and destabilize the surrounding atherosclerotic plaque. Then, the thrombus breaks off and travels through the blood circulation, being able to cause stroke or myocardial infarction [58,59].

### 3.3. Obesity, OS and Endothelial Blood Cells Function

**Figure 4:**
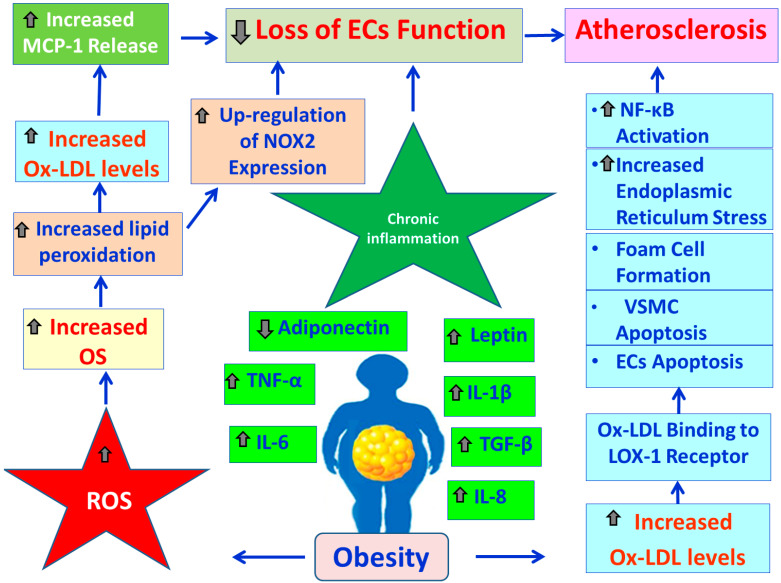
Illustration of the pathophysiological mechanisms involved in the development of atherosclerosis in obesity. In obesity, excess fatty tissue becomes an endocrine organ, releasing increased levels of pro-inflammatory cytokines like TNF-α, ΙL-1β, IL-6, IL-8, and TGF-β, as well as pro-inflammatory adipokines such as leptin. Concurrently, the levels of the anti-inflammatory adipokine adiponectin are reduced. This shift in secretion promotes chronic, low-grade inflammation causing EC dysfunction. Also, in obesity, excess generation of ROS can lead to increased OS, which damages lipids and causes lipid peroxidation. This process generates Ox-LDL and MCP-1. These molecules trigger a cascade of events causing EC dysfunction. In addition, the increased lipid peroxidation can up-regulate NOX2 expression, contributing to OS and inflammation, resulting in EC dysfunction. Also, increased Ox-LDL levels contribute to atherosclerosis by binding to LOX-1 on ECs and VSMCs. This binding triggers a cascade of events that induce (i) apoptosis in both cell types, (ii) formation of foam cells, (iii) ER stress, and (iv) NF-κΒ signaling activation, ultimately promoting the development of atherosclerotic plaques through cell death, inflammation, and plaque destabilization.

OS plays a crucial role in the oxidation of LDL particles, through the process of lipid peroxidation [60]. Accumulation of ox-LDLs in the arterial tunica intima initiates the start of atherosclerosis [58]. Moreover, high levels of ox-LDL can be caused by an increased oxidant capacity, which can result from elevated expression and activity of NOX2 [61,62]. Increased expression of NOX2 in turn promotes increased levels of pro-inflammatory cytokines, decreased production of adiponectin, and generation of ROS in blood vessels and immune cells, which can lead to further OS and development of atherosclerosis [63,64] (Figure 4). Another possible mechanism of the Ox-LDL implication in the development of atherogenesis involves the release of MCP-1 from the ECs and the VSMCs [65]. Specifically, Ox-LDL particles stimulate the secretion of MCP-1, which further facilitates the dysfunction of ECs and SMCs of the arterial media layer [66]. Loss of function of ECs provokes several cell surface adhesion molecules, such as connexin, Eph-ephrins, and Jagged-Notch3, which recruit monocytes from the blood into the endothelial space and these monocytes promote VSMC proliferation and vascular wall remodeling [67]. Monocytes then differentiate into macrophages that up-regulate both (i) TLFRs, which have a central role in macrophage activation and (ii) Srs, which remove apoptotic cell fragments, bacterial endotoxins and Ox-LDL. This leads to lipid accumulation and foam cell formation [68]. Then, cholesterol builds up in the inner lining of the artery, and an arterial plaque develops and forms a complex lesion with migration and proliferation of VSMCs, which secrete the ECM, such as collagen accumulation into the arterial plaques [69]. Atherosclerosis causes the expression of PDGF and TGF-β, which further increase VSMC proliferation and collagen deposition in the arterial plaque [67]. Activated macrophages lead to production of high levels of pro-inflammatory cytokines, proteases, and ROS, which promote atherosclerosis [68,70,71]. Also, it has been found that macrophages exposed to Ox-LDL up-regulate the expression of caveolin-1, which is important for the development of atherosclerosis [61,62]. However, EC-VSMC communication is bi-directional, meaning that changes occurring in VSMCs may ultimately affect ECs’ function. Suppression of VSMC proliferation causes elevated ER stress and NF-κB activation, which stimulate the release of factors, such as EDHF, Evs, and microRNAs, which leads to ECs apoptosis and subsequent atherosclerosis [70]. In addition, Ox-LDL can increase TG production by influencing both LPL [72] and the FA supply within adipocytes [73]. This increased lipotoxicity directly causes ECs dysfunction and subsequent atherosclerosis. Moreover, Ox-LDL binding to LOX-1 [74] stimulates (i) Ox-LDL uptake in both macrophages and SMCs, (ii) foam cell formation, and (iii) ECs activation [61,70]. It also causes EC-induced apoptosis of VSMCs [61,70]. In addition, the excessive formation of Ox-LDL induces an increased expression of Bax and Bcl-2, suggesting the involvement of LOX-1 in the atherosclerotic plaque destabilization [75,76]. Figure 4 demonstrates the possible mechanisms involved in atherosclerosis development by the Ox-LDLs.

### 3.4. Obesity, OS and Hypertension

**Figure 5:**
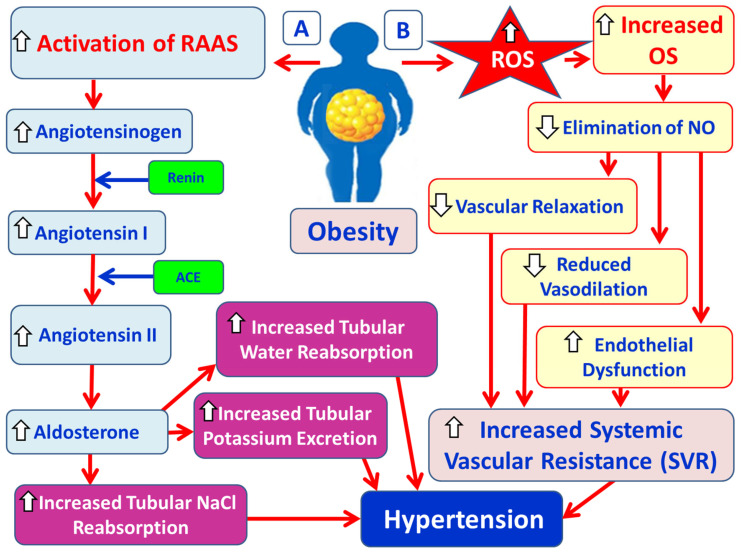

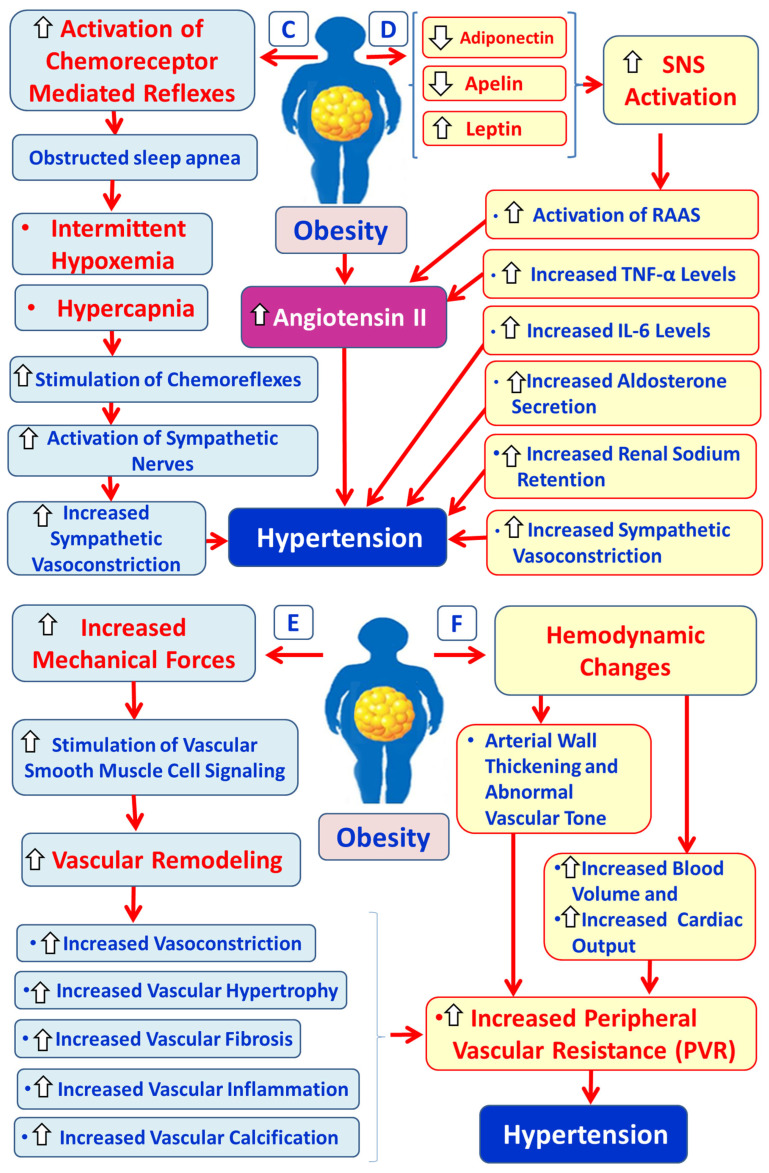
Illustration of the pathophysiological mechanisms involved in pathogenesis of obesity-associated hypertension. (**A**) Excess adipose tissue acts as an active endocrine organ activating the renin–angiotensin–aldosterone system (RAAS). Angiotensinogen forms angiotensin I under the action of renin. Angiotensin I works as a precursor of angiotensin II under the action of ACE. Angiotensin II stimulates aldosterone secretion from adrenal glands. Aldosterone increases sodium and water reabsorption, potassium excretion in the distal tubule and collecting duct of the kidneys resulting in hypertension. (**B**) Obesity promotes generation of free radicals and ROS through enzymes such as NOX, leading to an imbalance, which results in increased OS. Increased OS eliminates NO, decreasing vascular relaxation and vasodilation, resulting in SVR and hypertension. (**C**) Obstructive breathing events in obese patients are primarily characterized by OSA (obstructive sleep apnea) through the activation of chemoreceptor-mediated reflexes. OSA causes intermittent hypoxemia and hypercapnia, which promote activation of SNS, sympathetic vasoconstriction, and hypertension. (**D**) In obesity, the low adiponectin and apelin levels and the high levels of leptin are associated with the activation of SNS, causing activation of RAAS, increased levels of TNF-α and IL-6, and increased angiotensin II and aldosterone secretion, vasoconstriction, increased renal sodium retention, and hyperextension. (**E**) Obesity directly causes mechanical forces by creating mechanical stress-activating mechanosensors such as integrins, G proteins and ion channels on VSMCs, and stimulating VSMC signaling pathways. These events promote vascular remodeling with vasoconstriction, vascular hypertrophy, vascular fibrosis, vascular inflammation, and vascular calcification, which result in hypertension. (**F**) Obesity causes significant hemodynamic changes, leading to increased blood volume and cardiac output. In addition, obesity leads to arterial wall thickening and abnormal vascular tone, which refers to the prolonged constriction of the smooth muscles within the arterioles. These hemodynamic changes associated with obesity induce increased peripheral vascular resistance, which elevates blood pressure.

Hypertension is highly correlated with the MetS and predisposes to increased risk for CVDs [77,78]. Also, hyperinsulinemia and IR are found in hypertensive patients with T2DM [79]. Hypertension and obesity interact with cardiac function, leading to larger heart volume, left ventricular hypertrophy (Figure 5), and thus, greater likelihood of cardiac failure [79,80]. Specifically, increased SVR due to arterial wall thickening and abnormal vascular tone is the main hemodynamic situation in hypertension [77]. Multiple interacting factors, such as the activation of RAAS, OS, and SNS, hemodynamic changes, and mechanical forces, trigger signaling in VSMCs. This activation leads to processes such as vasoconstriction, vascular hypertrophy, fibrosis, inflammation, and calcification. These changes collectively constitute vascular remodeling, which causes increased arterial stiffness and contributes to the underlying problem of high blood pressure [77,81,82]. A free radical is the product of normal cellular metabolism, which has one or more unpaired electrons. A free radical is an atom or molecule with one or more unpaired electrons in its outer shell, making it highly reactive and unstable [83,84]. ROS such as superoxide anion radical (O_2_^−.^), hydroxyl radical (OH^.^), hydrogen peroxide (H_2_O_2_), and singlet oxygen (^1^O_2_) are highly reactive and unstable chemicals formed from diatomic oxygen (O_2_). Free radicals and other ROS are formed continuously in normal essential physiological processes [85]. OS occurs when there is an imbalance between the production of free radicals and ROS and the ability of the body to neutralize them with antioxidant defense [86]. High levels of ROS can directly damage DNA, proteins, and lipids [87]. LPO preferentially oxidizes PUFAs and causes structural modifications of lipids in plasma membranes [85]. An increase in free radicals and ROS eliminates nitrogen monoxide (NO), decreasing vascular relaxation and vasodilation, which results in an increase in the systemic vascular resistance (SVR) [83] (Figure 5). A decreased NO production, or an increased inactivation of NO because of its interaction with the superoxide anion radical (O_2_^−.^), or an imbalance between the superoxide (O_2_^2−^) and the NO production may account for reduced vasodilation and may promote endothelial dysfunction, leading to development of hypertension and cardiovascular complications [88,89].

#### 3.4.1. Physiology and Pathophysiology of RAAS

The RAAS plays an important role in the regulation of arterial BP. The RAAS interacts with the SNS and the baroreceptor reflexes [90]. In response to low BP, the SNS is the major determinant for conversion of prorenin to active renin from JG-cells within the afferent arterioles of the kidneys, via norepinephrine actions on the β1-adrenergic receptor-AMP pathway [91,92]. The enzyme renin, in the bloodstream, cleaves angiotensin I from angiotensinogen, a glycoprotein produced in the liver and fat cells [91]. Angiotensin I is a peptide hormone, physiologically inactive, and works as a precursor for angiotensin II [rapidly cleaved by ACE]. Angiotensin II is the physiological active component of the RAAS [90]. Angiotensin II has effects on the kidney, adrenal gland, arterioles, and brain by binding to angiotensin type I and angiotensin type II receptors, which are GPCR [93]. Angiotensin II causes vasoconstriction in renal and systemic arterioles by acting on VSMCs, increasing heart rate and contractility by activating the SNS. Therefore, angiotensin II increases SVR, and BP [91,94]. Also, angiotensin II acts on the ZG cells of the adrenal cortex and stimulates aldosterone secretion (Figure 5), which is a steroid hormone [95]. Aldosterone has multiple effects that regulate BP [96]. The fundamental role of aldosterone is to promote sodium and water reabsorption and potassium excretion in the kidneys’ distal tubule and collecting duct [91,97]. Moreover, aldosterone acts in the SNS and sensitizes the BP response to angiotensin II [98,99]. The pathological activation of RAAS leads to hypertension by causing excessive vasoconstriction [91]. Considerable evidence supports the role of the activation of the SNS in the pathogenesis of obesity-related hypertension [79,100]. Excessive adiposity increases BP by causing increased renal sodium reabsorption and impaired pressure natriuresis, meaning the kidneys require higher blood pressure to excrete sodium. This is due to physical compression from visceral fat, the activation of SNS, and increased activity of RAAS [101] (Figure 5). In obesity, the adipose tissue functions as an active endocrine organ, secreting components of the RAAS, including angiotensin II and various pro-inflammatory cytokines, such as IL-1β, IL-6 and TNF-α and adipokines, contributing to chronic, low-grade inflammation and IR. This inflammatory state directly connects adiposity to hypertension, MetS, and CVDs [77,102,103]. Particularly, increased angiotensin II levels enhance microvascular vasoconstriction by (i) reducing NO production, (ii) stimulating vasoconstrictors like ET-1 and prostanoids, (iii) promoting the contraction of VSMCs, and (iv) increasing SNS activity [104,105]. Angiotensin II induces OS through the activation of NOX enzymes and the production of ROS, such as superoxide anion radical (O_2_^−^) [106]. Also, angiotensin II induces lipid peroxidation, and the combination of increased LPO and reduced antioxidant status are connected to hypertension [107]. Moreover, angiotensin II stimulates the release of inflammatory cytokines [108], whilst increasing evidence indicates that the vital pathophysiological role of OS is in the development of hypertension, especially in obesity [109,110].

#### 3.4.2. Hypertension Due to Over-Activation of SNS in Obesity

Obesity is associated with increased SNS activity in tissues like the heart, kidneys, and skeletal muscles [111] (Figure 5). Especially, obese patients exhibit increased SNS activity, particularly in the kidneys and heart, which drives the development and maintenance of hypertension [112]. Several potential factors have been proposed to contribute to increased SNS activity in obesity. These factors include alterations in adipose tissue secretion of adipokines and cytokines. Specifically, low adiponectin levels as well as low apelin levels are associated with SNS activation and arterial hypertension [112,113,114]. Also, increased leptin secretion from adipose tissue activates SNS, leading to increased sodium and water retention by the kidneys, which elevates BP [112,115]. Moreover, sympathetic activation is related to increased levels of IL-6, which are implicated with increased BP, therefore linking systemic inflammation with an increased risk of hypertension [116]. In addition, TNF-α is essential for angiotensin II-induced hypertension [117]. Additionally, activation of the RAAS and the formation of angiotensin II are implicated. In obese individuals, significant amounts of angiotensin II are secreted from abdominal subcutaneous adipose tissue [118]. Also, adipocytes are capable of producing aldosterone, and this process is partially dependent on angiotensin II [119]. Increased angiotensin II levels due to adipose tissue dysfunction can increase SNS activity [104,105]. Angiotensin II signaling is enhanced in various brain sites, including the paraventricular nucleus (PVN), the rostral ventrolateral medulla (RVLM), and the area postrema, contributing to BP regulation and hypertension [120]. The RVLM is a key region in the brainstem, responsible for regulating arterial BP by controlling SNS activity [121]. The action of angiotensin II on SNS includes (i) activation of central adrenergic pathways, (ii) stimulation of sympathetic ganglia and the adrenal medulla, and (iii) neurotransmitter release from sympathetic nerve terminals, which can lead to increased vasoconstriction, increased sodium and water retention, and elevated heart rate, thereby raising BP [90,122]. Also, there is evidence that high aldosterone levels can increase BP by stimulating CNS to increase sodium appetite, sympathetic nerve activity, and vasopressin release, and impair pressoreflex (or baroreflex) sensitivity [104,123]. Dysfunction of the pressoreceptor reflexes may contribute to excessive RSNA in cases with obesity-related hypertension [100], whilst activation of chemoreceptor-mediated reflexes associated with OSA and intermittent hypoxemia may also be an important factor. Obstructive breathing events in obese patients cause repetitive events of hypoxemia and sometimes hypercapnia, which stimulate the central chemoreflex response, and in that way contribute to elevated blood pressure levels through SNS activation and sympathetic vasoconstriction [112,124,125].

## 4. Conclusions and Future Perspectives

Shared pathophysiological mechanisms between obesity, OS, ER stress, lipotoxicity, and inflammation are central to the pathogenesis of NAFLD and CVDs, working in a complex interplay where IR serves as a major driver [126,127,128]. OS is a shared underlying mechanism linking NAFLD and CVDs, as it fuels inflammation and cellular damage in both conditions [129,130,131,132]. In NAFLD, OS from mitochondrial dysfunction and lipid accumulation contributes to liver damage and the release of inflammatory factors [133,134,135]. In both NAFLD and CVDs, inflammation acts as a key driver and mediator of disease progression, fostering a systemic, low-grade inflammatory state [136,137,138,139]. For CVDs, inflammation disrupts endothelial function, promotes the growth of atherosclerotic plaques, and triggers blood clots [140,141]. Thus, inflammation creates a vicious cycle where NAFLD promotes systemic inflammation, which then fuels the development and progression of CVDs [142]. ER stress occurs in both NAFLD and CVDs through pathways like unfolded protein response (UPR) [143]. In NAFLD, excess lipids cause hepatic ER stress, leading to IR, inflammation, and liver injury [144]. This same cellular stress, along with systemic atherogenic dyslipidemia, systemic inflammation, and endothelial dysfunction, links NAFLD to an increased risk of CVDs [145]. Lipotoxicity, driven by dyslipidemia, leads to cellular injury, dysfunction, and death [146]. Lipotoxicity is a significant factor for the pathogenesis of NAFLD, particularly from non-alcoholic fatty liver (NAFL) to more severe NASH [147]. This toxic lipid accumulation triggers cellular stress, inflammation, and apoptosis, ultimately contributing to liver damage and, potentially, cirrhosis [146,148]. An understanding of these overlapping pathways opens new ways for early identification and targeted interventions of these conditions. However, in CVDs, there is the antioxidant paradox, which refers to the discrepancy where diets rich in antioxidants such as fruits and vegetables are associated with lower CVD risk, but large-scale clinical trials of non-specific antioxidant supplements like Vitamins E and C have shown little to no benefit, and sometimes increased mortality [149,150,151,152,153,154,155,156]. This paradox is not fully understood but may be due to (i) the complex synergy of nutrients in foods, (ii) the different processing by the body of antioxidant compounds in foods compared to that of antioxidant supplements, and (iii) the harmful effects of high doses of antioxidant supplements on the body [150,151,152]. In addition, there is an antioxidant paradox in NAFLD, which refers to the conflicting roles of antioxidants. Particularly, while low dietary antioxidant intake is linked to NAFLD, the effectiveness of antioxidant supplements remains unclear with some studies showing no benefit or even potential harm from high doses [157,158,159,160,161]. This antioxidant paradox in NAFLD may be due to the action of antioxidant compounds against normal levels of ROS, so that they are ultimately unable to exert their protective role in normal cellular signaling such as cell proliferation, differentiation, survival, and immune responses [157,158,159,160,161]. 

Future research should focus on further identification of specific biomarkers of oxidative damage that could aid in the stratification of individuals being at-risk and guide personalized therapies. Additionally, exploring the liver–adipose–nervous system axis and its influence on endothelial cell function may reveal novel therapeutic targets. These directions will ultimately lead to a multidisciplinary approach that integrates metabolic, inflammatory, and oxidative perspectives that will be essential for preventing and managing obesity-related NAFLD and CVDs more effectively.

## Data Availability

Not applicable.

## Abbreviations

AASLD: American Association for the Study of Liver Diseases; ACE: angiotensin-converting enzyme; Adipo-IR: Adipose tissue insulin resistance; AGEs: advanced glycation end products; APO-1: Fas death receptor; B-lps: apoB-containing lipoproteins; BOH: beta-hydroxybutyrate; BP: blood pressure; CEs: cholesteryl esters; CETP: cholesteryl ester transfer protein; CHD: coronary heart disease; CVDs: cardiovascular diseases; DNL: de novo lipogenesis; ECs: endothelial cells; ECM: extracellular matrix; EDHF: endothelium-derived hyperpolarizing factor; ET-1: endothelin-1; ER: endoplasmic reticulum; Evs: extracellular vesicles; FAs: fatty acids; FC: free cholesterol or unesterified cholesterol; FFAs: free fatty acids; GPCRs: G protein-coupled receptors; HCC: hepatocellular carcinoma; HL: hepatic lipase; HDL: high density lipoprotein; HSCs: hepatic stellate cells; HSL: hormone-sensitive lipase; HTG: hypertriglyceridemia; IL-1β: interleukin-1β; IL-6: interleukin-6; IL-8: interleukin-8; IR: insulin resistance; JG-cells: juxtaglomerular cells; LDs: lipid droplets; LDLs: low-density lipoproteins; LOX-1: Ox-LDL binds to lectin-like oxidized low-density lipoprotein receptor-1; LPL: lipoprotein lipase; LPO: Lipid peroxidation; MAFLD: metabolic-associated fatty liver disease; MASH: metabolic-associated steatohepatitis; MCP-1: monocyte chemoattractant protein 1; MetS: metabolic syndrome; NAFL: non-alcoholic fatty liver; NAFLD: non-alcoholic fatty liver disease; NASH: non-alcoholic steatohepatitis; NEFAs: non-esterified fatty acids; NF-κΒ: nuclear factor kappa B; NO: nitric oxide NOX enzymes: nicotinamide adenine dinucleotide phosphate (NADPH) oxidase; NOX2: nicotinamide adenine dinucleotide phosphate (NADPH) oxidase 2; OS: Oxidative Stress; OSA: obstructive sleep apnea; ox-LDLs: oxidized low-density lipoproteins; PL: phospholipids; PDGF: platelet derived growth factors; PLTP: phospholipid transfer protein; PUFAs: polyunsaturated fatty acids; RAAS: renin–angiotensin–aldosterone system; RCTs: randomized-controlled trials; ROS: reactive oxygen species; RSNA: renal sympathetic nerve activity; RVLM: rostral ventrolateral medulla; PVN: paraventricular nucleus; sdLDLs: small, dense LDLs; SMCs: smooth muscle cells; SNS: sympathetic nervous system; SRMAs: systemic reviews and meta-analyses; SRs: scavenger receptors; SVR: systemic vascular resistance; TCFA: thin-cap fibroatheroma; TGF-β: transforming growth factor-β; TLFRs: toll-like receptors; TNF-α: tumor necrosis factor-alpha; TAGs: triacylglycerols; TGs: triglycerides; TRLs: triglyceride-rich lipoproteins; T2DM: type 2 diabetes mellitus; ULDL: ultra low-density lipoprotein; UPR: unfolded protein response; VLDL: very low-density lipoprotein; VSMCs: vascular smooth muscle cells; ZG cells: zona glomerulosa (ZG) cells.
